# Insights in the Global Genetics and Gut Microbiome of Black Soldier Fly, *Hermetia illucens*: Implications for Animal Feed Safety Control

**DOI:** 10.3389/fmicb.2020.01538

**Published:** 2020-07-07

**Authors:** Fathiya M. Khamis, Fidelis L. O. Ombura, Komivi S. Akutse, Sevgan Subramanian, Samira A. Mohamed, Komi K. M. Fiaboe, Weerachai Saijuntha, Joop J. A. Van Loon, Marcel Dicke, Thomas Dubois, Sunday Ekesi, Chrysantus M. Tanga

**Affiliations:** ^1^Plant Health Theme, International Centre of Insect Physiology and Ecology, Nairobi, Kenya; ^2^Department of Integrated Pest Management, International Institute of Tropical Agriculture, Yaounde, Cameroon; ^3^Walai Rukhavej Botanical Research Institute (WRBRI), Biodiversity and Conservation Research Unit, Mahasarakham University, Maha Sarakham, Thailand; ^4^Laboratory of Entomology, Plant Sciences Group, Wageningen University, Wageningen, Netherlands

**Keywords:** genetic diversity, gut microbiome, *Hermetia illucens*, mitochondrial *COI* gene, 16S-metagenomics

## Abstract

The utilization of the black soldier fly (BSF) *Hermetia illucens* L. for recycling organic waste into high-quality protein and fat biomass for animal feeds has gained momentum worldwide. However, information on the genetic diversity and environmental implications on safety of the larvae is limited. This study delineates genetic variability and unravels gut microbiome complex of wild-collected and domesticated BSF populations from six continents using mitochondrial *COI* gene and 16S metagenomics. All sequences generated from the study linked to *H. illucens* accessions KM967419.1, FJ794355.1, FJ794361.1, FJ794367.1, KC192965.1, and KY817115.1 from GenBank. Phylogenetic analyses of the sequences generated from the study and rooted by GenBank accessions of *Hermetia albitarsis* Fabricius and *Hermetia sexmaculata* Macquart separated all samples into three branches, with *H. illucens* and *H. sexmaculata* being closely related. Genetic distances between *H. illucens* samples from the study and GenBank accessions of *H. illucens* ranged between 0.0091 and 0.0407 while *H. sexmaculata* and *H. albitarsis* samples clearly separated from all *H. illucens* by distances of 0.1745 and 0.1903, respectively. Genetic distance matrix was used to generate a principal coordinate plot that further confirmed the phylogenetic clustering. Haplotype network map demonstrated that Australia, United States 1 (Rhode Island), United States 2 (Colorado), Kenya, and China shared a haplotype, while Uganda shared a haplotype with GenBank accession KC192965 BSF from United States. All other samples analyzed had individual haplotypes. Out of 481,695 reads analyzed from 16S metagenomics, four bacterial families (Enterobactereaceae, Dysgonomonadaceae, Wohlfahrtiimonadaceae, and Enterococcaceae) were most abundant in the BSF samples. Alpha-diversity, as assessed by Shannon index, showed that the Kenyan and Thailand populations had the highest and lowest microbe diversity, respectively; while microbial diversity assessed through Bray Curtis distance showed United States 3 (Maysville) and Netherlands populations to be the most dissimilar. Our findings on genetic diversity revealed slight phylogeographic variation between BSF populations across the globe. The 16S data depicted larval gut bacterial families with economically important genera that might pose health risks to both animals and humans. This study recommends pre-treatment of feedstocks and postharvest measures of the harvested BSF larvae to minimize risk of pathogen contamination along the insect-based feed value chain.

## Introduction

The black soldier fly (BSF) *Hermetia illucens* (Linnaeus, 1758; Diptera: Stratiomiydae) is a highly adaptable saprophagous cosmopolitan insect species (Carles-Tolra and Andersen, 2002). Its distribution has widely expanded over time to the warmer parts of the world (Marshall et al., 2015). Global records of *H. illucens* indicate an increased frequency of encounters in Europe from 1950–1960, although this is not a true indication of their abundance. It was first recorded in southern Europe in 1926. Preceding these recordings, the first record of BSF in South Africa, Kenya, and Ghana were in 1915, 2015, and 2018, respectively (Picker et al., 2004; Marshall et al., 2015; Stamer, 2015; Chia et al., 2018). The exact original distribution of *H. illucens* is not well known. *H. illucens* was recorded in the Southeastern United States as far back as the 1800s (Marshall et al., 2015), reflecting a northward spread from a native range in Central America and the northern parts of South America in historical times. Several questions remain unanswered about the origin, invasion history and current distribution status of *H. illucens* because available data are scant. However, there is consensus that the spread of *H. illucens* was dependent on maritime transport that likely played a role in repeated, accidental introductions through trade of fruits and vegetables along coastlines and islands (Picker et al., 2004). Currently, molecular evidence supporting the biogeography of *H. illucens* and its colonization of Africa and the world at large is lacking.

Black soldier fly larvae are among the most efficient waste decomposers, recycling a wide range of organic waste into high-quality edible biomass of 38.5–62.7% crude protein and 14.0–39.2% fat content that is rich in energy (5282 kcal/kg gross energy; Sheppard et al., 1994; Tomberlin et al., 2005, 2009; Caruso et al., 2013; Banks, 2014; Myers et al., 2014; Lalander et al., 2015). The larvae are also a rich source of micronutrients (iron and zinc) and all essential amino acids including relatively high amounts of cereal-limiting amino acids such as lysine, threonine, and methionine. Processed larvae are therefore used as a high-quality protein valuable for feed ingredient for various monogastric animal species, including poultry, pigs and fish (Bondari and Sheppard, 1987; St-Hilaire et al., 2007). In addition, BSF is neither a pest nor a disease vector, and does not constitute a nuisance like other flies (Diener, 2010). Frass produced by BSF larvae is an excellent fertilizer able to increase crop yields. Finally, insect-based feed protein technologies, which can be implemented at low-cost, have the potential to provide employment opportunities and livelihood improvement for both farmers and urban entrepreneurs (Diener et al., 2015).

Despite the economic importance of *H. illucens*, currently no data is available on worldwide population genetics, including genetic variability within and between geographic populations. Knowledge of the genetic structure of BSF populations would provide a sound framework for gaining insight into their dispersion and mating compatibilities, and for identifying their actual and potential routes of gene flow. Also, lack of knowledge of the genetic structure of BSF populations have prevented identification of its areas of origin in newly colonized parts of the world, including Africa, tracing the route of its colonization process both within and outside North America, and assessment of colonization effects on population differentiation. The introduction or invasion of *H. illucens* has been reported in many countries with chances of the species undergoing rapid evolutionary events.

Bacteria are an essential component of decomposing organic waste (Burkepile et al., 2006; Barnes et al., 2010; Miki et al., 2010) and are always associated with insects such as BSF that use these resources. Many insect species including BSF largely depend on obligate bacterial mutualism for their survival, viability and reproduction (Werren et al., 1995). Recent efforts have demonstrated that BSF larvae reduce pathogenic bacteria within animal wastes (Erickson et al., 2004; Liu et al., 2008). Bacteria isolated from BSF larvae have been extensively used as probiotic to enhance manure reduction and subsequent larval development (Yu et al., 2011). Many of these beneficial bacteria could be natural constituents of the larval environment or potentially vertically transmitted. Although BSF might suppress potential pathogens, it is not clear if other opportunistic pathogens might proliferate in their presence and present potential health and environmental risks. In this study, genetic variability and microbial diversity among BSF populations from different geographic locations in the world were investigated using the barcode region of the mitochondrial cytochrome oxidase I (*mtCOI*) gene and microbiome through 16 S metagenomics.

## Materials and Methods

### Sampling

Larvae of BSF were collected from different indoor rearing facilities in various countries across the globe namely: Australia, China, Costa Rica, Ghana, Kenya, Nigeria, South Africa, Thailand, Netherlands, Uganda, and United States. The samples from each location were preserved in 95% ethanol and brought to the Arthropod Pathology Unit at the International Center of Insect Physiology and Ecology (*icipe*, Nairobi, Kenya) for further processing.

### DNA Extraction, Polymerase Chain Reaction (PCR) and Sequencing of the Insect Larvae

Each individual insect larva was surface sterilized using 3% NaOCl and rinsed with distilled water. Genomic DNA was extracted using the Isolate II Genomic DNA Kit (Bioline, London, and United Kingdom) following the manufacturer’s instructions. The purity and concentration of the resultant DNA was determined using a Nanodrop 2,000/2,000 c spectrophotometer (Thermo Fischer Scientific, Wilmington, United States). PCR was performed to amplify the *COI* barcode region of the mitochondrial DNA region in a total reaction volume of 20 μL containing 5X My *Taq* reaction buffer (5 mM dNTPs, 15 mM MgCl_2_, stabilizers, and enhancers; Bioline), 10 pmol/μl of primers [LepF1 5′ ATTCAACCAATCATAAAGATATTGG 3′, LepR1 5′ TAAACTTCTGGATGTCCAAAAAATCA 3′ (Hajibabaei et al., 2006)], 0.5 mM MgCl_2_, 0.0625 U μl^–1^ My *Taq* DNA polymerase (Bioline), and 15 ng/μl of DNA template in a Nexus Mastercycler gradient machine (Eppendorf, Hamburg, Germany). The following cycling conditions were used: initial denaturation for 2 min at 95°C, followed by 40 cycles of 30 s at 95°C, 45 s annealing at 52°C, extension for 1 min at 72°C, and a final elongation step of 10 min at 72°C. The amplified PCR products were resolved through a 1.2% agarose gel. DNA bands on the gel were analyzed and documented using KETA GL Imaging System *Trans*-Illuminator (Wealtec Corp, Meadowvale Way Sparks, United States). Successfully amplified products were excised and purified using Isolate II PCR and Gel Kit (Bioline) following the manufacturer’s instructions. Purified samples were shipped to Macrogen Europe BV (Meibergdreef, Amsterdam, Netherlands) for bi-directional sequencing.

### Next Generation Sequencing

Insect samples from each locality were surface sterilized in 3% NaOCl then washed thrice in sterile water. The cuticle was excised using a sterile scalpel and then the whole gut contents were transferred into 1.5 ml Eppendorf tubes from which genomic DNA was extracted as described above. The resultant DNA were lyophilized into 1.5 ml DNAstable tubes (Biomatrica, San Diego, United States) then sent to Macrogen Europe BV for 16 S metagenomics [Illumina 16 S amplicon (V3–V4 region) library preparation + Illumina MiSeq 2 × 300 bp sequencing, 100,000 reads per sample].

### Data Analyses

#### Mitochondrial DNA Data Analysis

The sequences obtained were assembled and edited using Chromas Lite Version 2.1.1^1^ and Geneious Version 8^2^ (Kearse et al., 2012). The primer sequences were identified and removed from the consensus sequences generated from both the forward and reverse reads. Pairwise and multiple alignments were performed in Clustal X software (version 2.1; Thompson et al., 1997). The evolutionary history was inferred by using the maximum likelihood method based on the Kimura 2-parameter model (Kimura, 1980) using MEGA X (Kumar et al., 2018). The tree with the highest log-likelihood (–1906.90) is shown. Initial tree(s) for the heuristic search were obtained automatically by applying Neighbor-Join and BioNJ algorithms to a matrix of pairwise distances estimated using the maximum composite likelihood (MCL) approach, and then selecting the topology with superior log-likelihood value. The reliability of the clustering pattern in the tree was evaluated using a bootstrap analysis with 1,000 replicates and involving 76 nucleotide sequences. Evolutionary divergence over sequence pairs between groups were calculated using the Kimura 2-parameter distance model (Kimura, 1980) in MEGA X and principal coordinate plots constructed from the genetic distances using GenAlEx 6.41 (Peakall and Smouse, 2006).

Bayesian analysis was carried out with MrBayes V.3.2 (Ronquist et al., 2012). The GTR + I + G model (general time reversible model incorporating variant sites and a gamma distribution) model was selected for Bayesian analysis as determined by MrModelTest V.2.3 (Nylander, 2008). In Bayesian analysis, the Markov chain Monte Carlo process was generated at four chains and 4,000,000 generations resulting in 40,000 trees. The sampling frequency was 100 generations. Analyses ran until the average standard deviation of split frequencies were below 0.01. The trees were checked for convergence of parameters (standard deviation of split frequencies and potential scale reduction factor) in MrBayes V.3.2. Effective sample size (ESS) was also checked using Tracer V1.5 (Rambaut and Drummond, 2007). The first 1,000,000 generations (10,000 trees) were excluded as the burn-in step, corresponding to the standard deviation of split frequencies below 0.01, potential scale reduction factor equal to 1.0 and ESS value above 200, indicating a good posterior probability distribution sample. The remaining trees (30,000 trees) were used to evaluate the posterior probabilities. The Bayesian topology was visualized using the FigTree V.1.4 program (Rambaut, 2012).

For conclusive identification of the species, similarity searches, phylogenetic analyses and genetic divergence of the *COI* barcoding gene were conducted. Similarity searches were carried out by querying the consensus sequences via the basic local alignment search tool (BLAST). The BLAST algorithm finds regions of local similarity between sequences, in which consensus sequences were compared to reference sequences in the GenBank database. All the sequences generated in the study were submitted to GenBank and assigned accession numbers (Table 1).

**TABLE 1:** Sample collection details and identification and GenBank accessions of *Hermetia illucens* from different regions.

**Country of collection**	**Collection points**	**Sample name**	**GenBank similarity and accession**	***E*-value**	**GenBank accession numbers assigned**
Australia (Tucki)	28° 46′ 13.79″ S, 153° 18′ 29.89″ E, 129 m.a.s.l	Aus 49–54	*Hermetia illucens*, FJ794367.1	0	MT483920 – MT483925
China (Pudang)	26° 07′ 48.4″ N, 119° 19′ 49.0″ E, 13 m.a.s.l	Chi 56–57, 59–60	*Hermetia illucens*, FJ794367.1	0	MT483914 – MT483917
Costa Rica (Escazu)	09° 55′ 15.06″ N, 84° 08′ 45.86″ W, 1087 m.a.s.l	CosR 1	*Hermetia illucens*, KC192965.1	0	MT483918
Ghana (Greater Accra)	05° 41′ 38.06″ S, 00° 01′ 58.93″ W, 298 m.a.s.l	Ghana 7–12	*Hermetia illucens*, FJ794361.1	0	MT483926 – MT483931
Kenya (Nairobi)	01° 13′ 14.6′′ S, 036° 53′ 44.5′′ E, 1612 m.a.s.l	Ken 31–36	*Hermetia illucens*, FJ794367.1	0	MT483937 – MT483942
Netherlands (Wageningen)	52° 08′ 28.15″ N, 05° 35′ 42.91″ E, 7 m.a.s.l	Neth 1–2, 4, 6–7	*Hermetia illucens*, KY817115.1	0	MT483932 – MT483936
Nigeria (Awka)	06° 22′ 18.60″ N, 07° 04′ 16.51″ E, 39 m.a.s.l	Nig 25–30	*Hermetia illucens*, FJ794361.1	0	MT520657 – MT520662
South Africa (Cape Town)	33° 55′ 14.79″ S, 18° 25′ 27.17″ E, 30 m.a.s.l	SA 1–6	*Hermetia illucens*, KY817115.1	0	MT520651 – MT520656
Thailand (Chiang Mai)	18° 56′ 04.63″ N, 98° 57′ 39.09″ E, 833 m.a.s.l	Thai 1–6	*Hermetia illucens*, KM967419.1 and FJ794355.1	0	MT520663 – MT520666; MT520687
Uganda (Kampala)	00° 21′ 08.99″ N, 32° 34′ 55.09″ E, 1186 m.a.s.l	Uga 19–24	*Hermetia illucens*, KC192965.1 and FJ794367.1	0	MT520675 – MT520680
United States (Rhode Island; Boulder; Maysville for United States 1, 2, and 3, respectively)	41° 34′ 48.34″ N, 71° 28′ 38.74″ W, 22 m.a.s.l; 39° 56′ 20.92″ N, 105° 10′ 37.67″ W, 2235 m.a.s.l; 38° 35′ 41.69″ N, 83° 47′ 05.69″ W, 254 m.a.s.l	United States _1: 37–42; United States _2: 15–18; United States _3: 44–46, 48	*Hermetia illucens*, FJ794367.1 and FJ794355.1	0	MT520681 – MT520686; MT520671 – MT520674; MT520667 – MT520670

#### BSF 16S Metagenome Data Analysis

Sample data consisted of pooled samples per location, comprising of 5 individuals from each of the 13 different geographical regions and 2,819 taxa. Illumina-sequenced paired-end fastq sequences were checked for quality using FastQC v 0.11.28 (Andrews, 2010) and pre-processed to remove adapters and sequencing primers using Cutadapt v1.18 (Martin, 2011). Illumina-sequenced paired-end fastq sequences were imported and assembled in QIIME2-2018.11 (Bolyen et al., 2019). The DADA2 pipeline (Callahan et al., 2016) was used to denoise the reads based on per base quality scores and merge the paired-end-reads the sequences into amplicon sequence variants (ASVs). Subsequently, the denoised representative sequences was checked for chimeric sequences using Qiime-Vsearch and the chimeric sequences filtered using Uchime and these were excluded from the downstream analyses. The resulting representative sequence set was aligned and given a taxonomic classification using SILVA 32 database^3^. Additional analyses, such as rarefaction curves and Good’s coverage, were carried out with QIIME2. During data filtering, a total of 77 low abundance features were removed based on prevalence. Data were normalized as described by Weiss et al. (2017). The bacterial reads were binned into OTUs using an open OTU-picking strategy with 97% similarity and taxonomic assignment against the SILVA 32 database, which uses a bacterial and archaeal classification based on Bergey’s Taxonomic Outlines (Boone and Mah, 2001; Garrity et al., 2005; De Vos et al., 2009; Krieg et al., 2012). Taxonomic composition was performed using stacked bar/area plot and a pie charts, a minimum abundance cut-off of 0.1% was used to select the most abundant taxa in each sample. Taxa with cumulative read counts below the 0.1% cut-off were collapsed into the “Others” category. Both alpha and beta diversity analyses were performed using the phyloseq package (McMurdie and Holmes, 2013) while Hierarchical Ward’s linkage clustering based on the Pearson’s correlation coefficient of the microbial taxa abundance was performed with the hclust function in the package stat generated with R version 3.4.3 (RStudio Team, 2015). In our study, the haplotype networks of the closely related species were constructed in R version 3.5.1 (RStudio Team, 2015).

## Results

### BSF Identification and Phylogeny

**FIGURE 1:**
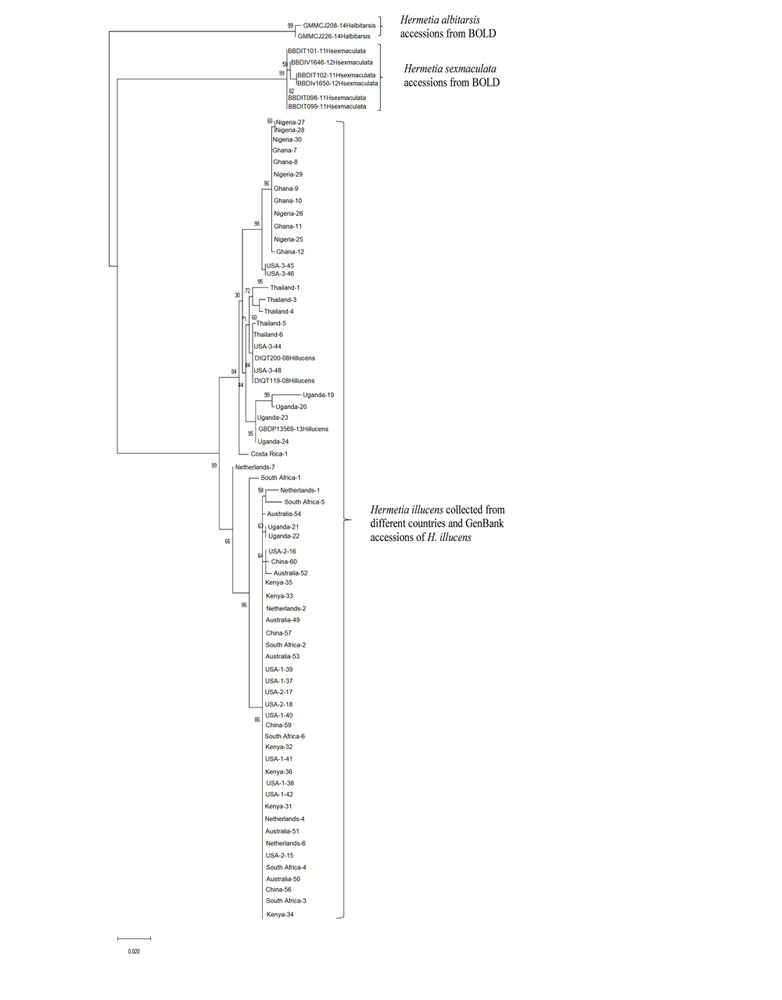
Phylogenetic relationship between for the *Hermetia illucens* samples collected from different countries and other GenBank accessions of closely related species inferred using Maximum Likelihood method by MEGA X (Kumar et al., 2018).

**FIGURE 2:**
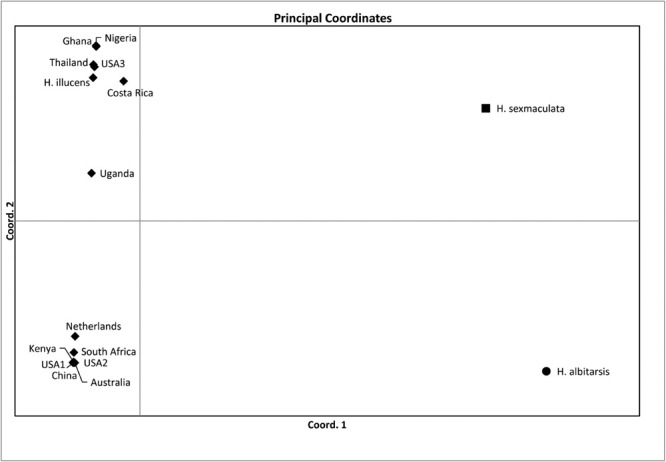
Plots of the principal coordinate analysis (PCoA) for the *Hermetia illucens* samples collected from different countries and other GenBank accessions of closely related species calculated using GenAlEx 6.4.

**FIGURE 3:**
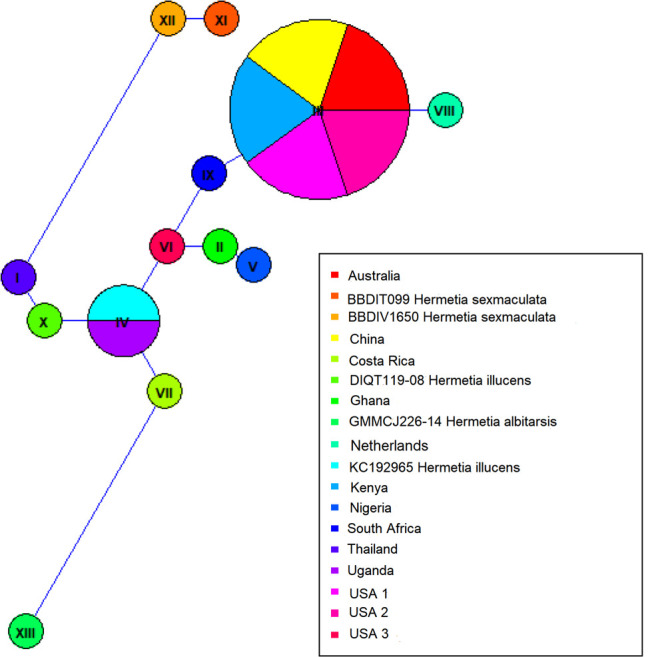
Haplotype network map of the BSF samples.

Both similarity and phylogenetic analyses were conducted for identification of species (Table 1). BLAST search linked all the sequences generated in the study to *H. illucens* accessions KM967419.1, FJ794355.1, FJ794361.1, FJ794367.1, KC192965.1, and KY817115.1 with a percentage IDs ranging from 97 to 100%. The tree separated all the samples into three branches, with a paraphyletic relationship between the *H. illucens* and *Hermetia sexmaculata* clusters. Samples from this study formed a monophyletic clade with *H. illucens* from GenBank. Within the *H. illucens* clade, the samples from West Africa (Nigeria and Ghana) formed a distinct cluster, samples from Thailand and United States 3 were closely related, while some samples from Uganda clustered separately. All samples from Australia, Netherlands, South Africa, Kenya, United States 1, United States 2, and China, clustered together (Figure 1). These results were further substantiated with the Bayesian analysis (Supplementary Figure 1). Estimates of evolutionary divergence over sequence pairs between groups were successfully generated from all sequenced samples and GenBank accessions of *H. illucens*, *H. sexmaculata* and *Hermetia albitarsis*. Numbers of base substitutions are presented as a genetic distance matrix (Table 2). The intraspecific genetic distance between *H. illucens* samples from the study and the GenBank accessions of *H. illucens* ranged between 0.91% and 4.07% which falls within the acceptable range of species limit. The *H. sexmaculata* and *H. albitarsis* samples clearly separated from all *H. illucens* by distances of 17.45% and 19.03%, respectively, confirming the phylogenetic analyses. The distance matrix was used to generate a Principal Coordinate Analysis (PCoA) where the first two axes in the PCoA plot explained 66.88% of the variation (the first axis 42.56% and the second axis 24.32%) between all the *Hermetia* samples analyzed from the study and GenBank accessions (Figure 2). The PCA clustered all *H. illucens* samples in the study with the GenBank accession of *H. illucens* in one axis though in specific clusters. However, that of two other species, *H. albitarsis* and *H. sexmaculata* occurred in the other axis, which further confirmed the phylogenetic analyses. The haplotype network map demonstrated that samples from Australia, United States 1, United States 2, Kenya, and China had a shared haplotype, while samples from Uganda shared a haplotype with a GenBank accession (KC192965) of *H. illucens* (Figure 3). The other samples each occupied an individual haplotype (Figure 3).

**TABLE 2:** Estimates of Evolutionary divergence over sequence pairs between groups generated by MEGA X (Kumar et al., 2018).

	**Hsexmaculata**	**Thailand**	**United States 3**	**Hillucens**	**Costa_ Rica**	**Uganda**	**Nigeria**	**Ghana**	**United States 2**	**SA**	**Australia**	**China**	**Kenya**	**Holland**	**United States 1**	**Halbitarsis**
Hsexmaculata	0.00%															
Thailand	17.74%	0.00%														
United States 3	17.37%	1.44%	0.00%													
Hillucens	17.33%	0.91%	1.07%	0.00%												
Costa_Rica	16.75%	1.88%	1.80%	1.46%	0.00%											
Uganda	17.89%	3.24%	3.16%	2.49%	3.32%	0.00%										
Nigeria	17.59%	2.73%	1.47%	2.28%	2.70%	3.97%	0.00%									
Ghana	17.55%	2.69%	1.43%	2.25%	2.66%	3.91%	0.10%	0.00%								
United States 2	17.47%	4.57%	4.12%	3.97%	4.54%	3.50%	4.83%	4.77%	0.00%							
SA	17.60%	4.66%	4.10%	4.07%	4.64%	3.69%	4.78%	4.74%	0.48%	0.00%						
Australia	17.45%	4.60%	4.17%	4.03%	4.59%	3.56%	4.88%	4.83%	0.16%	0.56%	0.00%					
China	17.41%	4.63%	4.17%	4.03%	4.60%	3.51%	4.88%	4.83%	0.12%	0.53%	0.21%	0.00%				
Kenya	17.41%	4.52%	4.06%	3.92%	4.49%	3.48%	4.78%	4.74%	0.05%	0.43%	0.13%	0.10%	0.00%			
Holland	17.33%	4.31%	3.94%	3.73%	4.16%	3.47%	4.69%	4.66%	0.61%	0.92%	0.68%	0.66%	0.56%	0.00%		
United States 1	17.41%	4.52%	4.06%	3.92%	4.49%	3.48%	4.78%	4.74%	0.05%	0.43%	0.13%	0.10%	0.00%	0.56%	0.00%	
Halbitarsis	21.34%	19.69%	19.11%	19.15%	17.70%	19.28%	19.86%	19.81%	18.79%	18.95%	18.73%	18.86%	18.73%	18.99%	18.73%	0.00%

*Distances were calculated as percentage of pairwise distances (*p*-distances) under the Kimura 2-parameter model.*

### BSF Bacterial Microbiota Diversity

**FIGURE 4:**
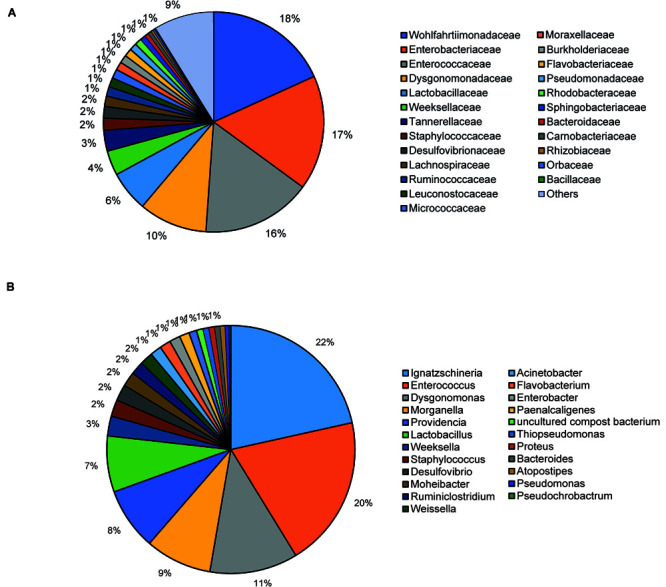
Taxonomic composition of bacterial community at **(A)** Family level and **(B)** Genus level using pie chart.

**FIGURE 5:**
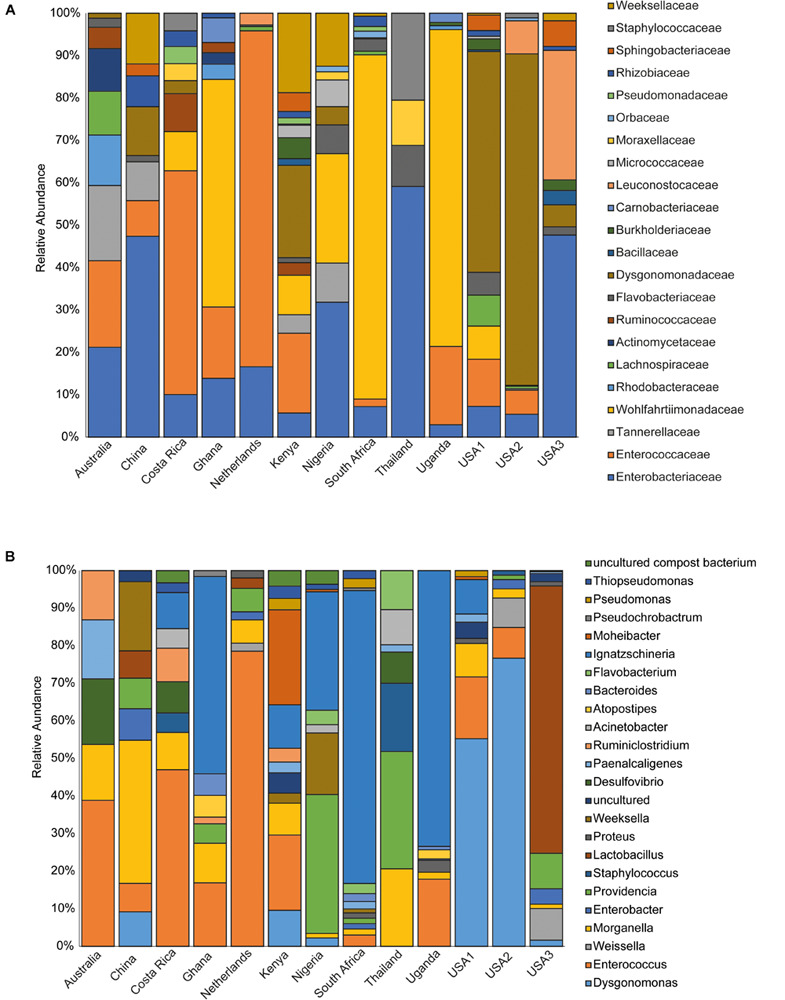
Incidence of the major bacterial taxonomic groups. The stacked bar chart shows the relative abundances of bacterial **(A)** Family and **(B)** genera identified in *Hermetia illucens* larvae analyzed from different countries.

**FIGURE 6:**
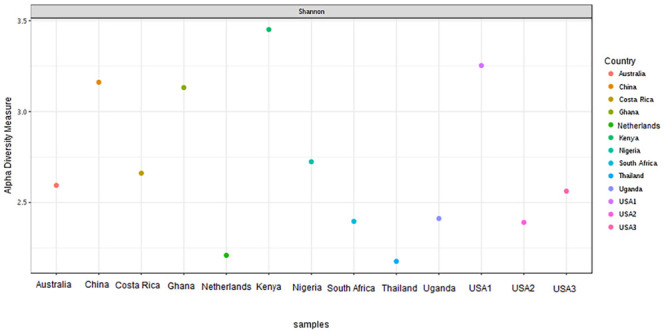
Alpha-diversity measure using Shannon at OTU level across all the samples. The samples are represented on *X*-axis and their estimated diversity on *Y*-axis. Each sample is colored based on country class.

**FIGURE 7:**
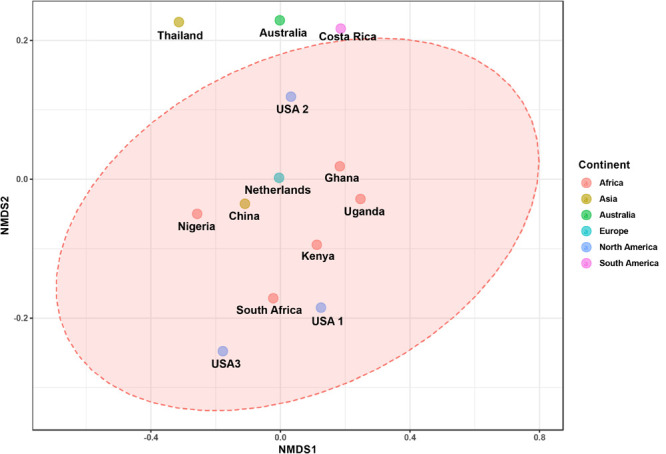
The ordination plot represented in 2-D NMDS plot using bray distance. Statistical significance is found out using (ANOSIM) *R*: 0.5558; *p*-value < 0.004.

**FIGURE 8:**
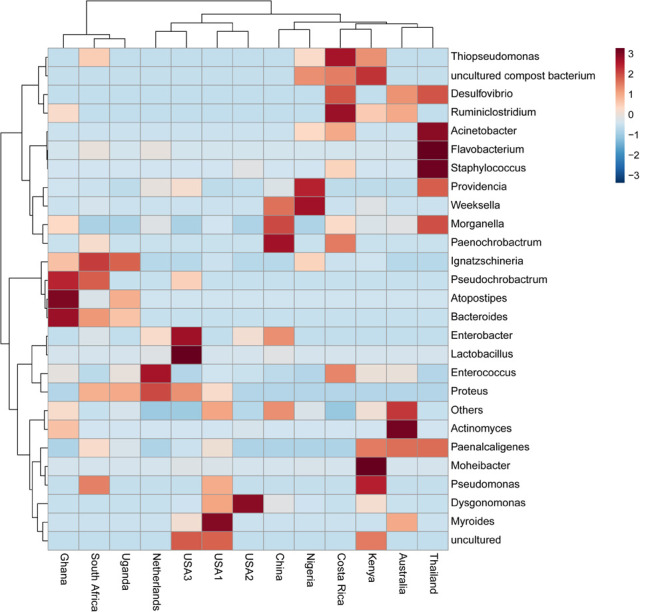
Heatmap based on microbiota composition at genus level. Hierarchical Ward’s linkage clustering based on the Pearson’s correlation coefficient of the microbial taxa abundance. Blue and red colors represent positive and negative correlations, respectively. The color scale represents the scaled abundance of each variable, denoted as Z-score, with red indicating high abundance, and blue indicating low abundance.

The bacterial microbiota analysis was based on a total of 188,863 sequences. The final dataset per sample was; Australia (16,840), China (14,766), Costa Rica (27,383), Ghana (10,376), Kenya (11,444), Netherlands (6,915), Nigeria (15,278), South Africa (12,658), Thailand (18,589), Uganda (8,973), United States 1 (12,199), United States 2 (19,263), and United States 3 (14,179). The direct quantitative comparison of the abundance of the microbiota showed the most abundant families were Wohlfahrtiimonadaceae (18.22%), Enterobacteriaceae (16.85%), Enterococcaceae (16.05%), and Dysgonomonadaceae (10.03%; Figure 4A). The comparison at genus level showed Ignatzschineria (22%), Enterococcus (20%), and Dysgonomonas (11%) to be the most abundant genera (Figure 4B). The cumulative abundance of the bacterial genera in the BSF populations showed that Enterobacteriaceae was the most abundant family in samples from Australia, China, Nigeria, Thailand and United States 3 while Dysgonomonadaceae was the most abundant in samples from Kenya, United States 1, and United States 2. Wohlfahrtiimonadaceae was the most abundant family in Ghana, South Africa and Uganda samples while Enterococcaceae was the most abundant in Costa Rica and Netherlands samples (Figure 5A). The most abundant genus in the samples from Australia, Costa Rica, and Netherlands was *Enterococcus*. Ghana, South Africa and Uganda had *Ignatzschineria* as the most abundant genus, Nigeria, and Thailand had *Providencia*, United States 1, and United States 2 had *Dysgonomonas*, China had *Morganella*, Kenya had *Moheibacter*, and United States 3 had *Lactobacillus* as the most abundant genus (Figure 5B). The intra population diversity (alpha diversity), as assessed by Shannon index, showed that the Kenyan population was the most diverse with a Shannon index of 3.5 while the Thailand population with a Shannon index of 2.1 had the least microbiome diversity (Figure 6). The microbial diversity between the populations as assessed through Bray Curtis distance showed the populations from United States 3 and Netherlands were the most dissimilar populations. Principal component analysis showed two main clusters: Australia, China, Ghana, Kenya, Nigeria, Thailand, Uganda, United States 1, and United States 2 in one cluster and Netherlands, South Africa, and Costa Rica in a second cluster (Figure 7). The hierarchical clustering as seen in the heatmap, showed the relative abundance of bacterial taxa within the populations of BSF analyzed in the study. The hierarchical linkage clustering based on Pearson’s correlation coefficient of the microbial taxa abundance showed three main clusters with Ghana, Uganda and South Africa similarly clustered, Australia, China, Costa Rica, Kenya, Nigeria, and Thailand similarly clustered while Netherlands, United States 1, United States 2, and United States 3 were similarly clustered. The correlation between the bacterial abundance and the source countries as shown in the quantitative comparison of the microbiota, indicated that the most abundant genus in each country was positively correlated to the source country in all the samples analyzed (Figure 8). All the 16S metagenomic data generated in this study has been submitted to GenBank and available through BioProject PRJNA625868.

## Discussion

It is believed that BSF has a common ancestry, and the phylogenetic analysis has shown very limited variation between the different populations based on mitochondrial *COI* barcode region. Common trade routes and similar feedstocks in these regions could be one of the reasons for this phenomenon. Furthermore, several studies have shown that other gene regions like Cytochrome b (*CytB*) gene (Chen et al., 2019) and NADH dehydrogenase 4 (*ND4*) gene (Tang et al., 1995) may be more polymorphic and thus give better resolution of intraspecific divergence of certain arthropod species. Therefore, an evaluation of the complete mitochondrial genome as well as other regions such as the nuclear microsatellites can be employed to resolve the population genetic structure of these closely related populations. The maximum likelihood and Bayesian analysis trees resulted in two branches of the BSF samples with clear separation of the two outgroups that were included in the analyses, *H. sexmaculata* and *H. albitarsis*. Despite the low genetic differences amongst the BSF populations in this study, five samples from four geographic regions (Australia, China, Kenya, and United States) shared a haplotype indicating a single origin for these populations. The Ugandan samples also shared a haplotype with GenBank accession of *H. illucens* from United States indicating the probable origin of the Uganda *H. illucens* sample. Two haplotypes were identified from the samples from West Africa (Nigeria and Ghana), but the variation between them was low and the phylogeny showed that they both also linked closely to a sample from the United States 3. The haplotype network also showed that the United States 3 haplotype was a centroid to the other haplotypes. Furthermore, the *H. illucens* samples from the study are closely related to samples from the United States as observed in the phylogeny and the haplotype network. Further research work comparing BSF samples from the native range in Northern South America countries such as Colombia, Venezuela, Guyana, Suriname, French Guiana, and Ecuador is crucial to establish the dispersal pattern from its native range to other parts of the world.

Based on the 16S rRNA sequencing and from the 481,695 reads analyzed, Enterobacteriaceae, Dysgonomonadaceae, Wohlfahrtiimonadaceae and Enterococcaceae were the most abundant families across the different countries. In addition, through taxonomic profiling, alpha-diversity (within-sample diversity) showed that the Kenyan and Thailand populations had the highest and the lowest microbiome diversity, respectively. Enterobacteriaceae, the most abundant family in Australia, China, Thailand, and United States 3, consists of bacterial genera that are ubiquitous in nature with many species free-living in diverse ecological niches, while some being associated with animals, plants or insects. Some of the species belonging to Enterobacteriaceae are significant human, animal, and plant pathogens causing a range of infections, hence the emphasis on sterile conditions for the rearing of the BSF. However, most of the species are not pathogenic and are utilized in several processes such as production of various recombinant proteins and non-protein products, control of infection diseases, anticancer agents, and biowaste recycling and bioremediation (Octavia and Lan, 2014). The family Dysgonomonadaceae was predominant in samples from Kenya, United States 1, and United States 2. Bacteria belonging to this family are capable of degrading various polysaccharides derived from host-ingested food, such as algae (Murakami et al., 2018) and could be an integral part in the role of BSF larvae as bioremediation organisms. Wohlfahrtiimonadaceae was the most abundant family in samples from Ghana, Nigeria and South Africa. This family consists of the genus *Wohlfahrtiimonas* which was described by Tóth et al. (2008) and consist of only one species, *Wohlfahrtiimonas chitiniclastica* S5^*T*^. This species has been associated with myiasis (Campisi et al., 2015) and so far, two cases of sepsis have been reported (Rebaudet et al., 2009; Almuzara et al., 2011). The most abundant family detected in the samples collected from Costa Rica was Enterococcaceae. This family is comprised of Gram-positive, facultatively anaerobic, anaerobic, or microaerophilic bacteria with some species being carboxyphilic or halophilic. They are associated with a wide range of ecological sources including; plants, humans, animals, the gastrointestinal tract of insects, fermented foods, drinking water, surface water, and seawater (Ludwig et al., 2009). The diverse bacterial genera identified from the different countries in this study could be indicative of the different diets in addition to the key BSF microbiota in the sampled countries with varying implications on the biology of the BSF as well as the industrial applications of BSF. For example, *Providencia*, which is vertically transmitted through the insect life cycle (De Smet et al., 2018) but can also be a pathogen in humans (Galac and Lazzaro, 2011) was most abundant in Nigeria and Thailand. Furthermore, BSF fed on fish diet has been shown to be exposed to gut dysbiosis because of a microbiota severely dominated by *Providencia* species (Bruno et al., 2019). *Ignatzschineria* which was the most abundant genus, in Ghana, South Africa and Uganda, has been shown to trigger repellency in *H. illucens* and thus reduce egg deposition (De Smet et al., 2018). *Dysgonomonas* which has been reported to degrade complex polysaccharides, was most abundant in two populations from United States (Bruno et al., 2019), and the high abundance of *Lactobacillus* in one population from United States has been shown to enhance biodegradation of food waste by *H. illucens* (Jiang et al., 2019).

Although the 16S data provides poor resolution at the species level, it has been able to unravel the families with genera that might pose risks to both animals and human health. As such the introduction of insects such as BSF as a high-quality protein ingredient in animal feed should be accompanied by proper safety measures. This postharvest treatment measures such as processing of the BSF larvae into dried products, defatting and proper storage in hermetic bags (Moreno-Martinez et al., 2000; Murdock et al., 2012) would be essential to minimize microbial spoilage and reduce the risk of pathogen contaminations along the insect-based feed value chain. Also, the choice of rearing substrate is crucial because of the putative transmission of microbiota to BSF larva with possible clinical implications. Although pre-treatment or sterilization of organic waste substrates before usage in all rearing systems across the world is a possible option, it is usually time-consuming and expensive. Therefore, we strongly recommend that harvested larvae of BSF from various waste substrates should be carefully sterilized during processing to eliminate potential microbial contaminants. Unfortunately, there are no published reports which provide evidence for the role of hygienic design, cooling facilities, sanitation programs and personal hygiene as measures to prevent microbial feed safety hazards for insect-based feed value chain. Therefore, potential preventive measures and intervention strategies as described above become crucial at all stages of the supply chain with thorough investigations in the insect-based protein feed enterprises globally.

## Data Availability Statement

The datasets presented in this study can be found in online repositories. The names of the repository/repositories and accession number(s) can be found in the article/ Supplementary Material.

## Author Contributions

CT and FK conceptualized the study. CT, FK, SE, FO, MD, KA, WS, and JL contributed to data curation. FK, CT, and FO did the formal analysis. MD, JL, SE, and CT acquired the funding. FK, CT, FO, KA, MD, JL, SE, KF, TD, SM, and SS carried out the investigation. FK, CT, and FO provided the methodology. MD, JL, SE, and CT contributed to project administration. MD, JL, CT, FK, and SE provided the resources. FK, FO, and CT helped with the software. CT, MD, JL, FK, SE, SS, and KF supervised the study. FK, CT, and FO validated the study and worked on the visualization. FK, FO, and CT wrote the original draft. FK, FO, KA, SS, SM, KF, WS, JL, CT, TD, MD, and SE reviewed, edited, and approved the final manuscript. All authors contributed to the article and approved the submitted version.

## Conflict of Interest

The authors declare that the research was conducted in the absence of any commercial or financial relationships that could be construed as a potential conflict of interest.

## Footnotes

1 https://technelysium.com.au/wp/chromas/

2 http://www.geneious.com

3 https://www.arb-silva.de/
