# Supplementary material for: Insights in the Global Genetics and Gut Microbiome of Black Soldier Fly, Hermetia illucens: Implications for Animal Feed Safety Control
Source: Front Microbiol. 2020 Jul 7;11:1538. doi: 10.3389/fmicb.2020.01538 (PMC7381391; doi:10.3389/fmicb.2020.01538)
Supplement: Supplementary file 1 [file Data_Sheet_1.docx]

Supplementary Material

# Supplementary Data


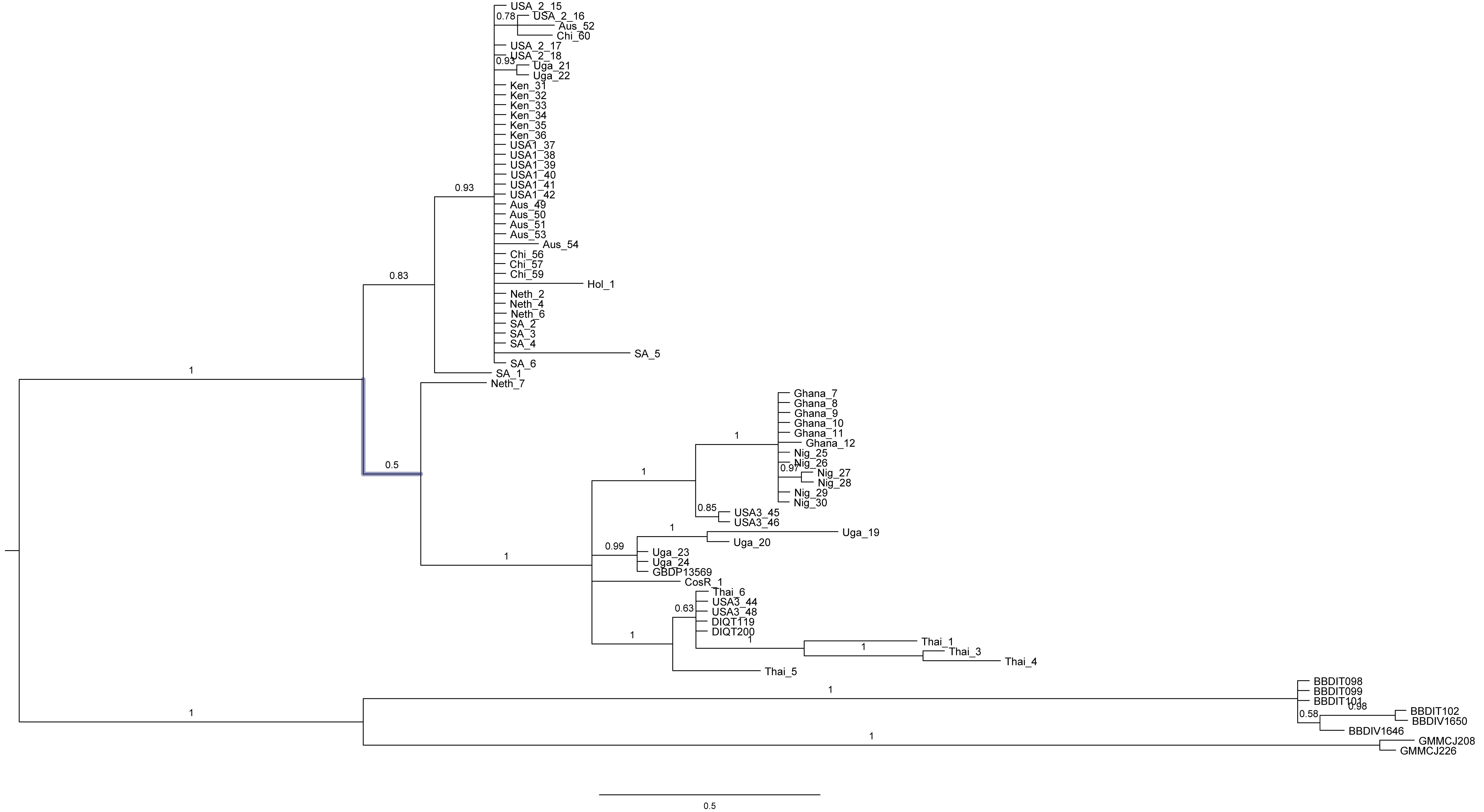


**Supplementary Figure 1.** Bayesian analysis tree topology and visualized using the FigTree V.1.4 program (Rambaut, 2012).
